# Dispersion engineering and frequency comb generation in thin silicon nitride concentric microresonators

**DOI:** 10.1038/s41467-017-00491-x

**Published:** 2017-08-29

**Authors:** Sangsik Kim, Kyunghun Han, Cong Wang, Jose A. Jaramillo-Villegas, Xiaoxiao Xue, Chengying Bao, Yi Xuan, Daniel E. Leaird, Andrew M. Weiner, Minghao Qi

**Affiliations:** 1 School of Electrical and Computer Engineering, Purdue University, West Lafayette, IN 47907 USA; 2 Birck Nanotechnology Center, Purdue University, West Lafayette, IN 47907 USA; 3 Purdue Quantum Center, Purdue University, West Lafayette, IN 47907 USA; 4 Facultad de Ingenierías, Universidad Tecnológica de Pereira, Pereira, RIS 660003 Colombia; 5 Shanghai Institute of Microsystem and Information Technology, Chinese Academy of Sciences, Shanghai, 200050 China; 6 Department of Electrical and Computer Engineering, Texas Tech University, Lubbock, TX 79409 USA; 7 Department of Electronic Engineering, Present Address: Tsinghua University, Beijing, 100084 China

## Abstract

Kerr nonlinearity-based frequency combs and solitons have been generated from on-chip microresonators. The initiation of the combs requires global or local anomalous dispersion which leads to many limitations, such as material choice, film thickness, and spectral ranges where combs can be generated, as well as fabrication challenges. Using a concentric racetrack-shaped resonator, we show that such constraints can be lifted and resonator dispersion can be engineered to be anomalous over moderately broad bandwidth. We demonstrate anomalous dispersion in a 300 nm thick silicon nitride film, suitable for semiconductor manufacturing but previously thought to result in waveguides with high normal dispersion. Together with a mode-selective, tapered coupling scheme, we generate coherent mode-locked frequency combs. Our method can realize anomalous dispersion for resonators at almost any wavelength and simultaneously achieve material and process compatibility with semiconductor manufacturing.

## Abstract (web summary)

Kerr frequency comb generation from microresonators requires anomalous dispersion, imposing restrictions on materials and resonator design. Here, Kim et al. propose a concentric racetrack-resonator design where the dispersion can be engineered to be anomalous via resonant mode coupling.

## Introduction

Recent advances on microresonators have realized Kerr frequency comb (or microcomb)^1–11^ and soliton^12–17^ generation on chip-scale photonic devices; high-quality factors (*Qs*) and the small modal volume of microresonators boost the Kerr nonlinearity and the free spectral ranges (FSRs) of resonant modes satisfy the required phase matching condition for the parametric four-wave-mixing processes. Mode-locked microcombs, especially those with formations of coherent solitons, provide a low noise and fine grid of comb lines, and have been applied to programmable photonic radio-frequency (RF) filters^18^, dual-comb spectroscopy^19^, microwave-to-optical links^20^, and massively parallel coherent optical communications^21^. To initiate the combs, anomalous dispersion is required for modulation instability^22–24^, and typical approaches^25, 26^ rely on the engineering of structural dispersions, which in many cases cause fabrication challenges, e.g., very thick films prone to cracking. Moreover, this approach fails in many spectral ranges such as the visible and ultraviolet where most materials show high normal dispersion.

Frequency combs at a relatively low normal dispersion regime have been reported previously^7–10^, and the general idea is that accidental mode coupling between different sets of resonating modes may cause strong but highly localized anomalous dispersion, allowing modulation instability. Such mode couplings have been observed between higher-order modes^7, 8^ or different polarizations^9^, but the accidental nature of the coupling makes the control of dispersion difficult. To control the mode interaction, dual-coupled resonators with thermal tuning have been introduced^10^, but the induced anomalous dispersion is still highly localized. Moreover, in the Si_3_N_4_ platform, which allows potential complementary metal–oxide–semiconductor (CMOS) integration^25^, these schemes still require thick Si_3_N_4_, which leads to significant film stress. Current methods to mitigate the stress rely on either deep trenches, which lead to difficulties in subsequent processes such as polishing and resist spinning^27, 28^, or the “photonic damascene” process which may cause issues in feature size and process control^29^. The use of compound-ring resonators to induce anomalous dispersion has been proposed with numerical simulation^30^. However, the proposed scheme is in general very sensitive to fabrication imperfections, making its realization difficult. In addition, high *Q*s and efficient pump coupling for the comb-generating mode also need to be achieved in order to generate frequency combs in such compound-ring resonators.

In this work, we present a concentric racetrack resonator design that can engineer the resonator dispersion with a high degree of control, and experimentally demonstrate strong anomalous dispersion and subsequently coherent frequency comb generation. To prove the concept of overcoming the material dispersion limit, we chose a thin (300 nm) Si_3_N_4_ platform, which shows high normal dispersion in the near-infrared (IR). An adiabatically tapered concentric racetrack resonator allows for selective excitation of the mode that shows anomalous dispersion, and coherent mode-locked Kerr frequency combs are demonstrated.

## Results

### Dispersion engineering with a concentric racetrack resonator

**Fig. 1:**
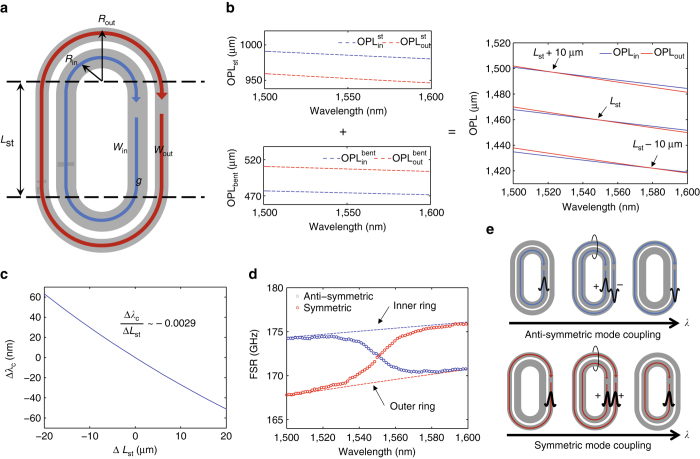
Optical path length engineering with a concentric racetrack resonator. **a** Schematic of a concentric racetrack resonator. The device height *h* is set to be 300 nm, and other geometric parameters are *R*_out_=50 μm, *g*=900 nm, *w*_in_=2,750 nm, *w*_out_=1,200 nm, and *L*_st_ =300 μm. **b** Optical path length (OPL) engineering of inner (OPL_in_, *blue*) and outer (OPL_out_, *red*) resonant modes. Note that at the straight section, the OPL of the inner racetrack is longer than the OPL of the outer racetrack $\left( {{\rm{OPL}}_{{\rm{st}}}^{{\rm{in}}} \,  >\, {\rm{OPL}}_{{\rm{st}}}^{{\rm{out}}}} \right)$ while it is opposite at the bending area $\left( {{\rm{OPL}}_{{\rm{bent}}}^{{\rm{in}}} \, < \, {\rm{OPL}}_{{\rm{bent}}}^{{\rm{out}}}} \right)$. The change in the straight section *L*_st_, e.g., ±10 μm, results in a slight shift of the wavelength (*λ*_c_) at which OPL matches. **c** Quantitative shift in OPL matching wavelengths with different *L*_st_. **d** Calculated free spectral ranges (FSRs) of anti-symmetric (*blue circles*) and symmetric (*red circles*) resonant modes. *Blue* and *red dashed lines* are the FSRs of inner and outer resonators, respectively, when there is no mode coupling. **e** Illustrations of anti-symmetric and symmetric mode couplings

Figure 1a gives the schematic of a racetrack-type concentric resonator showing two fundamental TE modes, ${\rm{TE}}_0^{{\rm{in}}}$ (*blue*) and ${\rm{TE}}_{\rm{0}}^{{\rm{out}}}$ (red). With the geometric parameters listed in the caption of Fig. 1, the mode overlap is negligible in both the straight and curved sections, and in the absence of resonant coupling, the modes reside mainly in the inner and outer racetracks, respectively. However, when the round-trip optical path lengths OPL_in_ and OPL_out_ of the inner and outer racetracks are matched (OPL_in_=OPL_out_), or in other words, the phase matching condition is met, there is nontrivial crosstalk between inner and outer modes exchanging their optical paths, and this process leads to resonant mode coupling. Figure 1b shows the detailed OPL matching between inner and outer resonators with OPLs at bent (OPL^bent^) and straight (OPL^st^) sections. The OPL_in_ and OPL_out_ can be represented as the following,

1 $$\begin{array}{ccccc}{\rm{OP}}{{\rm{L}}_{{\rm{in}}}} & = {\rm{OPL}}_{{\rm{in}}}^{{\rm{st}}} + {\rm{OPL}}_{{\rm{in}}}^{{\rm{bent}}}\\ & = 2{L_{{\rm{st}}}}n_{{\rm{in}}}^{{\rm{st}}} + 2\pi {R_{{\rm{in}}}}n_{{\rm{in}}}^{{\rm{bent}}}\end{array}$$

2 $$\begin{array}{ccccc}{\rm{OP}}{{\rm{L}}_{{\rm{out}}}} & = {\rm{OPL}}_{{\rm{out}}}^{{\rm{st}}} + {\rm{OPL}}_{{\rm{out}}}^{{\rm{bent}}}\\ & = 2{L_{{\rm{st}}}}n_{{\rm{out}}}^{{\rm{st}}} + 2\pi {R_{{\rm{out}}}}n_{{\rm{out}}}^{{\rm{bent}}}\end{array}$$

where *L*_st_ is the length of the straight section and *R* is the effective bending radius of each mode. The *n* is the effective refractive index of each waveguide mode (calculated with Lumerical Solutions Inc. www.lumerical.com), and there are four different *n* values: the inner and outer rings at both the straight and bent areas. In general, at the bending area, ${\rm{OPL}}_{{\rm{out}}}^{{\rm{bent}}}$ is greater than ${\rm{OPL}}_{{\rm{in}}}^{{\rm{bent}}}$ due to the larger bending radius, while at the straight section, ${\rm{OPL}}_{{\rm{in}}}^{{\rm{st}}}$ is greater than ${\rm{OPL}}_{{\rm{out}}}^{{\rm{st}}}$ due to the larger *n* with a larger waveguide width. This opposite OPL difference (ΔOPL) allows us to match the round-trip OPLs at desired wavelengths almost independently of the coupling strength. The OPL matching point (wavelength) can be engineered by changing the *L*_st_, and Fig. 1c shows the shift in OPL matching wavelengths, $\Delta {\lambda _c}{\rm{/}}\Delta {L_{{\rm{st}}}}\sim - 0.0029$. This independent and very fine control of the mode coupling point distinguishes our method from the scheme proposed in ref. ^30^, where phase matching between two concentric rings, i.e., without the straight sections, was to be achieved by varying the waveguide widths, which requires precise width control at sub-10 nm precision, and is much more difficult than achieving the right *L*_st_. Varying the *L*_st_ also changes the FSR of the device, and to achieve an arbitrary FSR and the desired wavelength region of anomalous dispersion together, further precise optimization is required in geometries such as bending radii and gap size.

As a result of the exchange in optical paths between the inner and outer rings, the resonant mode coupling generates symmetric (*ω*_s_) and anti-symmetric (*ω*_a_) hybridized modes, and their resonant frequencies can be represented by

3 $${\omega _{{\rm{a,s}}}} = \frac{{{\omega _{\rm{m}}} + {\omega _{\rm{n}}}}}{2} \pm \sqrt {\frac{{{{\left( {{\omega _{\rm{m}}} - {\omega _{\rm{n}}}} \right)}^2}}}{4} + \kappa _\omega ^2} ,$$

where *κ*_*ω*_ is the coupling coefficient and *ω*_m_ and *ω*_n_ are the resonant modes at the inner and outer racetracks without mode coupling, which can be found by OPL_in_= m*λ*_m_ and OPL_out_ = n*λ*_n_ (m,n: real integers). The positive and negative signs correspond to *ω*_a_ and *ω*_s_, respectively. The calculated FSRs of each mode are plotted in Fig. 1d, where the FSR of the anti-symmetric mode (*blue circles*) decreases as the wavelength increases. This indicates anomalous dispersion in the anti-symmetric mode $\left( {{D_\lambda } = \frac{\partial }{{\partial \lambda }}\left( {\frac{1}{{{\rm{FS}}{{\rm{R}}_\nu }(\lambda )L}}} \right)  >0} \right)$. Note that the dispersion of the inner and outer racetrack modes in spectral regions away from the resonant mode coupling, which are asymptotic to the dashed lines, are both normal (positive slopes in FSR). The anomalous dispersion comes only from the resonant mode coupling. An intuitive understanding of these FSR curves is that the anti-symmetric mode evolves from the inner racetrack to the outer one as the wavelength increases, while it is opposite for the symmetric mode (Fig. 1e). We note that the exact dispersion at each segment of the resonator is not critical as long as there is an overall balance of the OPL of the two modes (or the accumulated round-trip phase delays are matched).

### Experimental demonstration of resonant mode coupling

**Fig. 2:**
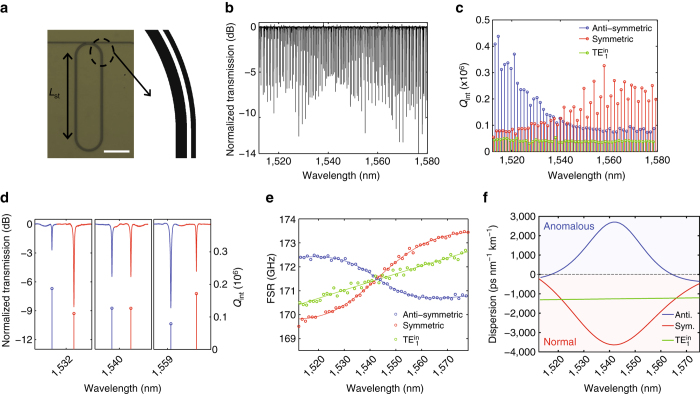
Resonant mode coupling in a concentric racetrack resonator. **a** Optical image and zoomed-in layout of the concentric racetrack resonator (*scale bar*: 100 μm). The nominal geometric parameters are the same as in Fig. 1. **b** Normalized transmission spectrum and **c** fitted intrinsic quality factors (*Q*_int_) of each mode: anti-symmetric (*blue*), symmetric (*red*), and first-order TE mode at inner ring ${\rm{TE}}_{\rm{1}}^{{\rm{in}}}$ (*green*). **d**, Zoomed-in view of **b** before, at, and after mode coupling. **e** Characterized free spectral range (FSR) of each mode (*solid lines*: fitting curves). **f** Characterized dispersion of each mode

Figure 2a shows the optical image of the fabricated device and Fig. 2b shows the normalized transmission (black). Figure 2c is the characterized intrinsic quality factors (*Q*_int_) assuming under-coupling, which scale inversely with propagation losses. There are three families of resonant modes; we can assign them as anti-symmetric (*blue*), symmetric (*red*), and ${\rm{TE}}_{\rm{1}}^{{\rm{in}}}$ (*green*) based on the difference between their trends of *Q*_int_ vs. wavelength. First, the mode that has relatively low and constant *Q*_int_ is assigned to ${\rm{TE}}_{\rm{1}}^{{\rm{in}}}$ (note that *w*_out_=1200 nm is too narrow to support the TE_1_ mode), which means that the round-trip loss of the ${\rm{TE}}_{\rm{1}}^{{\rm{in}}}$ mode does not change appreciably with the wavelength, a reasonable assumption when the mode profile and optical path do not change drastically within the wavelength range. The round-trip losses, or *Q*_int_, of the other two modes, however, change significantly in the measured wavelength range. According to Fig. 1e, the anti-symmetric mode at shorter wavelengths is well confined in the inner racetrack, which is much wider than the outer one. In general, a wider waveguide offers higher field confinement and yields higher *Q*_int_ due to the reduction of scattering losses from the sidewall roughness. Therefore, the *Q*_int_ is larger at shorter wavelengths and decreases as the wavelength increases since the anti-symmetric mode (shown in *blue* in Fig. 2c) evolves to the outer racetrack, which has a smaller width. Meanwhile, the opposite trend is observed for the symmetric mode (shown in *red* in Fig. 2c). Figure 2d is the zoomed-in view of a few resonant modes in Fig. 2b and shows that the extinction ratios of the resonances also exhibit opposite trends vs. wavelength. For the anti-symmetric mode, the on-resonance extinction increases with wavelength, suggesting an increase of coupling between the bus waveguide and the resonator in the under-coupled regime. This is consistent with our determination that the anti-symmetric mode evolves from the inner racetrack to the outer one. Again, the opposite trend is observed for the symmetric mode.

After unequivocally determining the mode families, we characterized the FSRs and dispersions of each mode in Fig. 2e, f, respectively. The ${\rm{TE}}_{\rm{1}}^{{\rm{in}}}$ (*green*) mode has high normal dispersion (around −1200 ps nm^−1^ km^−1^), which is a typical dispersion value for 300 nm thick Si_3_N_4_ resonators that prevents frequency comb generation. The FSRs for the symmetric (*red*) and anti-symmetric (*blue*) modes clearly show different trends, with the desirable anomalous dispersion occurring for the anti-symmetric mode and normal dispersion for the symmetric mode. The measured FSRs are qualitatively consistent with the simulated ones shown in Fig. 1d; in particular, the resonant mode-coupling point, or the wavelength at which OPL_in_= OPL_out_, matches fairly well. This is important as the anomalous dispersion band should reside within the gain bandwidth of erbium-doped fibre amplifiers (EDFAs) to have access to strong optical pumping.

### Selective mode excitation and frequency comb generation

For microcomb generation, in addition to the dispersion requirements, a higher *Q*_int_ (> 10^6^) to build up optical power within the cavity is also necessary. We increased the bending radius and racetrack waveguide widths to reduce the scattering losses from the sidewall roughness. Using a fabrication method we recently developed^11^, we were able to achieve high intrinsic *Q*s in the 1–2 million range, and we observed frequency comb generation (see Supplementary Fig. 1). While this comb has reasonable bandwidth (~180 nm), it has a highly uneven spectrum shape and high RF beating noise, which are both characteristics of type-II combs^5, 6^. Consequently, such combs may not be attractive for applications.

**Fig. 3:**
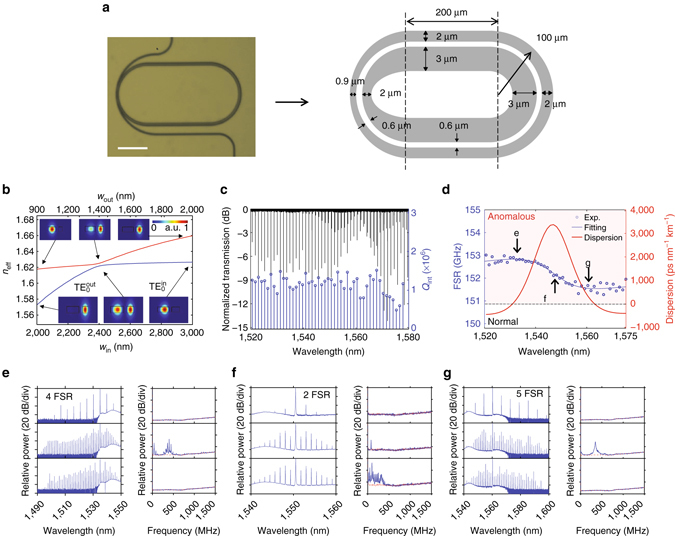
Frequency comb generation with a concentric racetrack resonator. **a** Optical image (*scale bar*: 100 μm) and zoomed-in layout (not to scale) of the concentric racetrack resonator. The film thickness *h* remains 300 nm, *R*_out_=100 μm, *g*=600 nm, *L*_st_=200, *w*_in_=3,000 nm, and *w*_out_=2,000 nm (except in the tapered portion of the device). The curved section on the left side of the resonator is tapered so that the widths of the inner and outer rings change adiabatically from *w*_in_=3,000 to 2,000 and then back to 3,000 nm, and *w*_out_=2,000 to 900 and then back to 2,000 nm, respectively. The gap between the inner and outer bends remains constant at *g*=600 nm. **b** Simulated effective refractive indices through the tapered section (*blue*: anti-symmetric mode, *red*: symmetric mode). *Insets* show the mode profiles at each section of tapering. **c** Normalized transmission spectrum (*black*) and fitted intrinsic quality factors (*Q*_int_) of the anti-symmetric mode (*blue*). **d** Free spectral range (*blue*) and corresponding dispersion (*red*) of the anti-symmetric mode. *Arrows* indicate the spectral positions of the optical pumping in **e**–**g**. **e**–**g** Evolutions of comb spectra and RF noises with pumping at different resonant modes of **e** *λ*_p_ ∼1,535.6 nm, **f** *λ*_p_ ∼1,550.6 nm, and **g** *λ*_p_ ~1,560.3 nm, respectively. The pump laser is tuned into resonance by red-shifting the pump wavelengths. The pump powers are **e** 425 mW, **f** 495 mW, and **g** 485 mW at the bus waveguide. The *red lines* on the RF measurements are the background noise of the electronic spectrum analyzer (ESA)

Our phase-matching condition is highly tolerant and requires only that there be an overall balance of optical path length for the two resonant modes. Therefore, we can adiabatically taper the region of the resonator that couples to the bus waveguide to excite the anti-symmetric mode selectively (Fig. 3a, also see Methods for detailed geometry) within a certain wavelength range. According to Fig. 1e, in order to evolve into the anti-symmetric mode, the mode should be concentrated in the inner racetrack (${\rm{TE}}_{\rm{0}}^{{\rm{in}}}$-like) at wavelengths shorter than the mode coupling point. However, due to the proximity of the outer racetrack to the bus waveguide, power will always be coupled to the outer racetrack with much higher efficiency. Therefore, the taper should transform the mode from the outer racetrack (${\rm{TE}}_{\rm{0}}^{{\rm{out}}}$-like) to the inner one (TE$_0^{\rm{in}}$-like). Figure 3b shows the simulated effective refractive indices of the designed adiabatically tapered concentric racetrack bends (at *λ*_0_=1,550 nm). Insets show the mode profiles at three different pairs of *w*_in_ and *w*_out_. With this tapering, the pump light is first coupled to the ${\rm{TE}}_{\rm{0}}^{{\rm{out}}}$ mode due to the proximity of the outer racetrack to the bus waveguide. Then, the ${\rm{TE}}_{\rm{0}}^{{\rm{out}}}$ mode transforms into the ${\rm{TE}}_{\rm{0}}^{{\rm{in}}}$ mode as light propagates through the tapered section, following the *blue line* in Fig. 3b. Our tapering design also provides a mode filtering function to prevent the excitation of other unnecessary higher-order and symmetric modes^16, 31^. Figure 3c shows the transmission spectrum and *Q*_int_ of the anti-symmetric mode. As expected, compared to Fig. 2b, the spectrum shows almost a single resonant mode at wavelengths shorter than the OPL matching wavelength (~1,548 nm). Figure 3d shows the characterized FSR (*blue*) and dispersion (*red*) of the dominant resonant mode. Notice the anomalous dispersion that is due to anti-symmetric coupling at the single-mode regime (1,530–1,548 nm). At longer wavelengths, two mode families exist and we chose the resonance dips that form a smooth dispersion spectrum (Fig. 3d, *red curve*) when combined with the dispersion derived from the single-mode regime (< 1,548 nm). This suggests that our resonator design allows the excitation of the anti-symmetric mode at wavelengths longer than the OPL matching point (supported by comb generation shown in Fig. 3g), even though the tapers favour the symmetric mode which manifests itself as the deep resonance dips in the wavelength range of 1,566–1,580 nm. The average *Q*_int_ does not show trends similar to Fig. 1b, suggesting that the *Q*_int_ is limited by the introduction of the tapers in the coupling region. Despite that, a high *Q*_int_ around 1 × 10^6^ is achieved across the measurement range.

To generate combs, we used the conventional method of laser tuning that slowly scans from blue to red. We pumped at three representative resonances: at wavelengths shorter than (1,535.0 nm), at (1,550.6 nm), and longer than (1,560.4 nm) the wavelength of peak anomalous dispersion, and Fig. 3e–g show the corresponding comb spectra, respectively. Low-frequency RF noise spectra are plotted next to the comb spectra. The comb lines expand predominantly to the shorter wavelength regime when we pump at 1,535.0 nm (Fig. 3e), while they expand predominantly to the longer wavelength regime when we pump at 1,560.4 nm (Fig. 3g). In both cases pumping occurs at wavelengths with close to zero dispersion, and the comb expands asymmetrically, extending mainly into the region with small and approximately wavelength-independent normal dispersion. A mode-locking transition, in which a noisy comb switches to a low-noise phase-locked state^32^, was also observed in both combs. The low RF noise in the combs suggests high coherence, which is confirmed by line-by-line shaping and autocorrelation measurements (see Supplementary Fig. 2)^6^. On the other hand, the microcomb in Fig. 3f, where pumping occurs in the resonant mode coupling region with large anomalous dispersion, shows very different behaviour. Here, the comb exhibits a symmetric profile with very limited bandwidth, and it is restricted to the region where the dispersion is large, anomalous, and strongly wavelength dependent. Furthermore, this comb exhibits high RF noise, and the autocorrelation data (see Supplementary Fig. 2) indicate poor compressibility under line-by-line shaping, a hallmark of incoherence.

### Mode-locking transition under single and dual pumping

Given the improved CMOS compatibility of our concentric resonators, it is worthwhile to explore a potential path for comb excitation with on-chip tunable laser sources. Compound semiconductors can provide an array of tunable lasers, but the output power of each laser is limited. A potential solution is to pump the resonator with multiple lasers at different FSRs of the resonator, as it has been reported that bi-chromatic pumping^33–37^ can reduce the required pump power and help maintain the coherence of the combs.

**Fig. 4:**
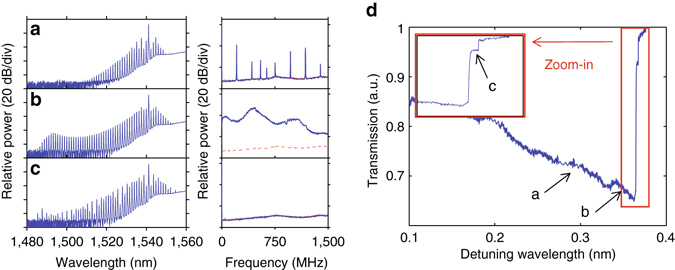
Mode-locking transition with single pump. **a**–**c** Evolution of comb spectrum and corresponding RF intensity noise (*blue*: signal, *red*: background noise) with a forward tuning. **d** Power transmission at the through port while scanning the resonance (∼1,542.2 nm) with a single-pump laser from *blue* to *red*. *Inset* shows the zoomed-in view of the transmission and *arrows* indicate each stage that corresponds to comb evolution

We first use a standard single-wavelength pump scheme that slowly scans the resonance from blue to red (forward or red tuning) around 1,542.2 nm. This wavelength is selected because it has been shown that pumping at a wavelength with an anomalous dispersion that is commensurate with the cavity decay rate helps initiate single-FSR combs^5, 7, 38^. Figure 4a–c show the evolution of comb spectra and RF noises. The first initiated side band was 2 FSR away but a very slight detuning resulted in a one-FSR comb as shown in Fig. 4a. The comb bandwidth then expands (Fig. 4b) and with further detuning the comb enters a low-RF-noise state (Fig. 4c). Figure 4d records the transmitted power while we scan the resonance, and the detuning at which we observed combs in Fig. 4a–c are marked. The *inset* in Fig. 4d shows that corresponding to the comb transition into the low-RF-noise state, there is a discrete jump in the transmitted power and is a strong indication of soliton generation based on previous reports^12–14^. The intensity modulation on the comb spectrum suggests that it might be a multi-soliton state, though a more detailed time domain characterization, e.g., an autocorrelation or FROG (frequency-resolved optical gating) measurement covering a large portion of the comb spectrum, is needed to confirm the formation of soliton(s).

We then introduce two pump lasers, one fixed in the highly anomalous dispersion regime at ${\lambda _{{\rm{p}}2}}\sim 1,543.4$ nm (Agilent TLS 8164), the other scanned approximately 1 FSR away (${\lambda _{{\rm{p}}1}}\sim 1,542.2$ nm) (New Focus TLB-6700). We note the scanning wavelength is the same as we showed in Fig. 4a–c. Since on-chip laser sources are not likely to be phase locked between each other, the two lasers in our experiments are free running, similar to those used in ref. ^35^, i.e., there are fluctuations of wavelength, power, and phase between the two lasers. The outputs of the two lasers were combined and fed into the same EDFA for amplification. The overall bi-chromatic pump power used in this comb generation is ~0.55 W in the bus waveguide of our resonator with a combined threshold power of ~0.1 W for comb initiation, lower than comb generation in the single-pump power case (~0.7 W with a threshold power of ~0.2 W).

**Fig. 5:**
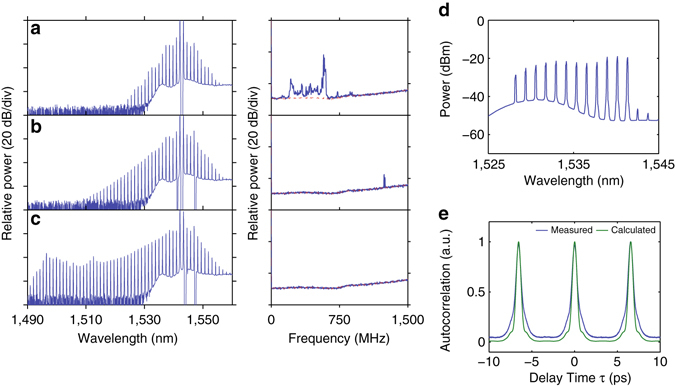
Mode-locking transition with bi-chromatic pump. **a**–**c** Evolution of comb spectrum and corresponding RF noise (*blue*: signal, *red*: background noise) with bi-chromatic pumping at two resonances separated by one free spectral range: ${\lambda _{{\rm{p}}1}}\sim 1,542.2$ nm (scanning) and ${\lambda _{{\rm{p}}2}}\sim 1,543.4$ nm (fixed). **a**–**c** are at slightly detuned *λ*_p1_ while scanning from *blue* to *red*. **d** Spectrum for autocorrelation after the pulse shaper and amplifier. **e** Autocorrelation of the comb from **d** with fibre dispersion compensated via the pulse shaper (*blue*) and autocorrelation computed assuming experimental spectrum with flat spectral phase (*green*)

Figure 5a–c show the evolution of the frequency comb under a bi-chromatic pumping with two free-running lasers. When scanning *λ*_p1_ from *blue* to *red*, the comb transitions from a narrow bandwidth one with high-RF noise (Fig. 5a) slowly to a broader bandwidth one with lower-RF noise (Fig. 5b) and then into a low-RF-noise state with the broadest bandwidth (Fig. 5c). Further detuning will again lead to narrower bandwidth and high-RF noise. We note that in the single-pump scenario, where the comb with a smooth spectral envelope (Fig. 4b) has high-RF noise, and the one having low-RF noise has significant modulation on the spectrum envelope (Fig. 4c). With bi-chromatic pumping, however, one could achieve a smooth comb spectrum envelope as well as low-RF noise. A portion of the comb spectrum in Fig. 5c (shown in Fig. 5d) is selected for autocorrelation measurements (see Methods), and the result (Fig. 5e) suggests that it forms a single pulse with a repetition period of 6.56 ps corresponding to the FSR of the resonator (152.4 GHz). Due to the limited spectral bandwidth out of which the pulse is formed, we do not claim the formation of a single soliton over the entire comb spectrum, but this autocorrelation measurement is strong evidence for mode locking of the comb under bi-chromatic pumping scheme. We note that recent analysis and simulations showed that bi-chromatic pumping, especially when the two pump lasers have similar intensity, which is the case in our experiment, can generate cavity solitons in both dispersion regimes—bright solitons in the anomalous dispersion regime^37^ and dark solitons in the normal dispersion regime^36^. The results above suggest that one could integrate the concentric resonators with an array of on-chip laser sources to achieve a monolithic, coherent frequency comb source that is amenable to volume manufacturing.

## Discussion

In summary, we present a complete set of design guidelines to simultaneously achieve well-controlled phase matching of two resonating modes in a concentric racetrack resonator; resonant mode coupling to significantly alter the FSR and to achieve anomalous dispersion with the anti-symmetric mode; and preferential coupling to the anti-symmetric mode and filtering out the symmetric and other higher-order modes. These guidelines are robust and tolerant enough to allow us to experimentally demonstrate concentric racetrack resonators with intrinsic *Q*s over 1 × 10^6^ on a 300 nm thick Si_3_N_4_ platform, which is more amenable than the previous thick (700–1,000 nm thick) Si_3_N_4_ films for fabrication in a commercial CMOS foundry. Despite the high normal dispersion in 300 nm thick Si_3_N_4_ waveguides, we successfully generated coherent mode-locked Kerr frequency combs in the near-IR. Therefore, our new resonators exhibit many important phenomena of microcombs in the anomalous dispersion regime, albeit with a comb bandwidth smaller than some of the recent demonstrations, i.e., octave spanning in the near-IR^17, 39, 40^ and near octave spanning in the mid-IR^41, 42^. The bandwidth of our resonator also can be broadened via further dispersion engineering. To achieve broadband frequency combs, near-zero anomalous dispersion is desirable^43^, and in our resonator scheme, this can be done by reducing the coupling strength, which can be engineered by the gap distance between the two resonators. We want to point out that in many applications such as telecommunications and microwave photonics, limiting the comb bandwidth might be preferred since it could boost the energy of individual comb lines. Moreover, our methodology should allow microcombs to be generated in many spectral ranges that are currently not feasible due to high normal material dispersion, e.g., visible and ultraviolet (there still exists other challenges such as material absorption and resonator *Q* in these frequency regimes). Therefore, we expect our methodology and demonstration to have considerable impact for on-chip frequency comb research and to pave the way for potential practical applications because the fabrication appears more feasible than before in a commercial CMOS foundry without the latest lithography capability.

## Methods

### Device fabrication

A <100>-oriented 4-inch silicon wafer was used as a substrate, and a 3 μm-thick silicon dioxide was thermally grown in a wet oxidation furnace. A 300 nm thick stoichiometric Si_3_N_4_ film was deposited by a low-pressure chemical vapour deposition (LPCVD). The concentric racetrack resonator was patterned by a 100 kV electron-beam (e-beam) lithography tool with hydrogen silsesquioxane (HSQ) negative tone e-beam resist. The nitride film was etched by an inductively coupled plasma reactive ion etcher (ICP-RIE) with CHF_3_ and O_2_. The HSQ was removed by a buffered oxide etcher to avoid the influence of the e-beam resist. The patterned device was annealed at 1,150 °C for 3 h in N_2_ ambient gas. A 3 μm-thick silicon dioxide top cladding was deposited as a low-temperature oxide in an LPCVD furnace. Using photolithography, the edges of the inverse taper fibre couplers were defined and etched through the substrate for 90 μm with ICP-RIE. The fibre edge coupler efficiency is 6 dB/facet.

### Device geometry

All the device heights *h* are set to be 300 nm, and other geometric parameters for the device in Figs 1 and 2 are *h*=300 nm, *R*_out_=50 μm, *g*=900 nm, *w*_in_=2,750 nm, *w*_out_=1,200 nm, and *L*_st_=300 μm. The bus waveguide width is 1.2 μm, and the gap between the bus and outer ring is 300 nm. For the device in Fig. 3 the parameters are *h*=300 nm, *R*_out_=100 μm, *g*=600 nm, *L*_*st*_=200 nm, *w*_in_=3,000 nm, and *w*_out_=2,000 nm (in the untapered portion of the device). The region of the device with coupling to the bus waveguide (left side of Fig. 3a) is tapered so that the widths of the inner and outer rings change adiabatically from *w*_in_=3,000 to 2,000 to 3,000 nm and *w*_out_=2,000 to 900 to 2,000 nm, respectively. The gap size *g* between inner and outer rings is held constant at *g*=600 nm. The arc length over which the taper occurs is 300.22 μm for the inner ring and 311.96 μm for the outer ring. The bus waveguide width is 1.2 μm, the gap between bus and outer ring is 200 nm, and the bending radius of bus waveguide is 130 μm.

### Pulse shaping and time domain autocorrelation measurements

As described in ref. ^6^, we selected a few comb lines and conducted line-by-line phase correction to form a transform-limited pulse. The compressed pulse was amplified with an EDFA to compensate for the losses from the pulse shaper, then measured with an autocorrelator based on noncollinear second-harmonic generation. The agreement of the compressed signal and calculated signal with flat phases (calculated based on the intensity of the comb lines in the optical spectrum) indicates that the pulse is close to bandwidth limited^6^. The same measurement setup was used for the autocorrelation in Fig. 5e, but without any phase correction on the comb lines; here the pulse shaper was used to compensate only for the dispersion of the fibre link and the measurement system. For dispersion compensation, a mode-locked fibre laser was used as a reference. These methods of using a pulse shaper to form a bandwidth-limited pulse and to compensate for the dispersion of the link have been widely used in previous research and verified with other methods^6, 8, 44, 45^.

### Data availability

The data that support the findings of this study are available from the corresponding author on request.

## Electronic supplementary material

Supplementary Information

## References

[1] Kippenberg TJ, Holzwarth R, Diddams S (2011). Microresonator-based optical frequency combs. Science.

[2] DelHaye P (2007). Optical frequency comb generation from a monolithic microresonator. Nature.

[3] Razzari L (2010). Cmos-compatible integrated optical hyper-parametric oscillator. Nat. Photon..

[4] Levy JS (2010). Cmos-compatible multiple-wavelength oscillator for on-chip optical interconnects. Nat. Photon..

[5] Herr T (2012). Universal formation dynamics and noise of Kerr-frequency combs in microresonators. Nat. Photon..

[6] Ferdous F (2011). Spectral line-by-line pulse shaping of on-chip microresonator frequency combs. Nat. Photon..

[7] Liu Y (2014). Investigation of mode coupling in normal-dispersion silicon nitride microresonators for Kerr frequency comb generation. Optica.

[8] Xue X (2015). Mode-locked dark pulse Kerr combs in normal-dispersion microresonators. Nat. Photon..

[9] Ramelow S (2014). Strong polarization mode coupling in microresonators. Opt. Lett..

[10] Xue X (2015). Normal-dispersion microcombs enabled by controllable mode interactions. Laser Photon. Rev..

[11] Xuan Y (2016). High-*q* silicon nitride micro-resonators exhibiting lowpower frequency comb initiation. Optica.

[12] Herr T (2014). Temporal solitons in optical microresonators. Nat. Photon..

[13] Wang P-H (2016). Intracavity characterization of micro-comb generation in the single-soliton regime. Opt. Express.

[14] Guo H (2016). Universal dynamics and deterministic switching of dissipative Kerr solitons in optical microresonators. Nat. Phys..

[15] Brasch V (2016). Photonic chip-based optical frequency comb using soliton cherenkov radiation. Science.

[16] Kordts A, Pfeiffer M, Guo H, Brasch V, Kippenberg T (2016). Higher order mode suppression in high-q anomalous dispersion sin microresonators for temporal dissipative Kerr soliton formation. Opt. Lett..

[17] Li Q (2017). Stably accessing octave-spanning microresonator frequency combs in the soliton regime. Optica.

[18] Xue X (2014). Programmable single-bandpass photonic rf filter based on Kerr comb from a microring. J. Lightw. Technol..

[19] Suh M-G, Yang Q-F, Yang KY, Yi X, Vahala KJ (2016). Microresonator soliton dual-comb spectroscopy. Science.

[20] Del’Haye P (2016). Phase-coherent microwave-to-optical link with a self-referenced microcomb. Nat. Photon..

[21] Marin-Palomo P (2017). Microresonator-based solitons for massively parallel coherent optical communications. Nature.

[22] Matsko AB, Savchenkov AA, Strekalov D, Ilchenko VS, Maleki L (2005). Optical hyperparametric oscillations in a whispering-gallery-mode resonator: threshold and phase diffusion. Phys. Rev. A..

[23] Matsko AB, Savchenkov AA, Ilchenko VS, Seidel D, Maleki L (2012). Hard and soft excitation regimes of Kerr frequency combs. Phys. Rev. A.

[24] Hansson T, Modotto D, Wabnitz S (2013). Dynamics of the modulational instability in microresonator frequency combs. Phys. Rev. A.

[25] Moss DJ, Morandotti R, Gaeta AL, Lipson M (2013). New Cmos-compatible platforms based on silicon nitride and hydex for nonlinear optics. Nat. Photon..

[26] Jung H, Poot M, Tang HX (2015). In-resonator variation of waveguide cross-sections for dispersion control of aluminum nitride micro-rings. Opt. Express.

[27] Nam KH, Park IH, Ko SH (2012). Patterning by controlled cracking. Nature.

[28] Luke K, Dutt A, Poitras CB, Lipson M (2013). Overcoming Si3N4 film stress limitations for high quality factor ring resonators. Opt. Express.

[29] Pfeiffer MH (2016). Photonic damascene process for integrated high-q microresonator based nonlinear photonics. Optica.

[30] Soltani M, Matsko A, Maleki L (2016). Enabling arbitrary wavelength frequency combs on chip. Laser Photon. Rev..

[31] Huang, S.-W. *et al*. Smooth and flat phase-locked Kerr frequency comb generation by higher order mode suppression. *Sci. Rep*. **6**, 26255 (2016). 10.1038/srep26255 PMC4867630 27181420

[32] Liang W (2014). Generation of a coherent near-infrared Kerr frequency comb in a monolithic microresonator with normal GVD. Opt. Lett..

[33] Papp SB, DelHaye P, Diddams SA (2013). Parametric seeding of a microresonator optical frequency comb. Opt. Express.

[34] Liu, Y. *et al*. Dual-pump generation of on-chip combs with low intensity noise. In *Frontiers in Optics*, FM4E-3 (Optical Society of America, 2013).

[35] Strekalov DV, Yu N (2009). Generation of optical combs in a whispering gallery mode resonator from a bichromatic pump. Phys. Rev. A.

[36] Lobanov V, Lihachev G, Kippenberg T, Gorodetsky M (2015). Frequency combs and platicons in optical microresonators with normal GVD. Opt. Express.

[37] Hansson T, Wabnitz S (2014). Bichromatically pumped microresonator frequency combs. Phys. Rev. A.

[38] Wang, C. *et al*. Mid-infrared optical frequency combs at 2.5 μm based on crystalline microresonators. *Nat. Commun*. **4**, 1345 (2013). 10.1038/ncomms2335 PMC3562444 23299895

[39] Okawachi Y (2011). Octave-spanning frequency comb generation in a silicon nitride chip. Opt. Lett..

[40] DelHaye P (2011). Octave spanning tunable frequency comb from a microresonator. Phys. Rev. Lett..

[41] Luke K, Okawachi Y, Lamont MR, Gaeta AL, Lipson M (2015). Broadband mid-infrared frequency comb generation in a si 3 n 4 microresonator. Opt. Lett..

[42] Yu M, Okawachi Y, Griffith AG, Lipson M, Gaeta AL (2016). Mode-locked mid-infrared frequency combs in a silicon microresonator. Optica.

[43] Okawachi Y (2014). Bandwidth shaping of microresonator-based frequency combs via dispersion engineering. Opt. Lett..

[44] Supradeepa V, Leaird DE, Weiner AM (2009). Optical arbitrary waveform characterization via dual-quadrature spectral interferometry. Opt. Express..

[45] DelHaye, P. *et al*. Phase steps and resonator detuning measurements in microresonator frequency combs. *Nat. Commun*. **6**, 5668 (2015). 10.1038/ncomms6668 25565467

