# Supplementary material for: Dispersion engineering and frequency comb generation in thin silicon nitride concentric microresonators
Source: Nat Commun. 2017 Aug 29;8:372. doi: 10.1038/s41467-017-00491-x (PMC5575100; doi:10.1038/s41467-017-00491-x)
Supplement: Supplementary file 1 — Supplementary Information [file 41467_2017_491_MOESM1_ESM.pdf]

Description of Supplementary Files

File Name: Supplementary Information
Description: Supplementary Figures and Supplementary References

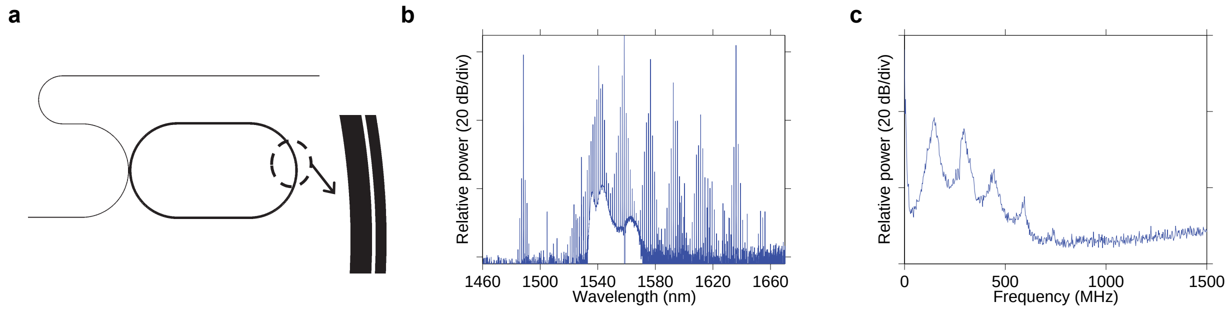

Supplementary Figure 1. **Type II frequency comb with a concentric-racetrack-resonator.** **a,** Layout image of the concentric-racetrack resonator, whose bending radius and widths are increased to $R_{\text{out}} = 100\ \mu\text{m}$, $w_{\text{out}} = 1400$ nm, and $w_{\text{in}} = 2800$ nm, respectively. Other geometric parameters are height $h = 300$ nm, gap $g = 500$ nm, and straight section length $L_{\text{st}} = 150\ \mu\text{m}$. To increase the coupling efficiency between bus waveguide and resonators, we bent the bus waveguide with the same bending radius of $R = 100\ \mu\text{m}$. **b,** Generated Type II comb spectrum and corresponding **c,** Radio-frequency (RF) intensity noise. The generated comb exhibits non-native mode spacing (a type II comb), with high RF intensity noise attributed to beating between sub-combs [1, 2].

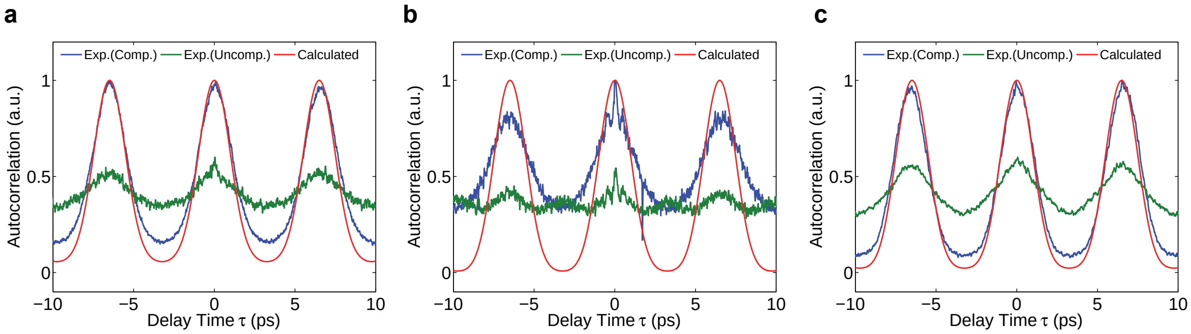

Supplementary Figure 2. **Line-by-line pulse shaping and autocorrelation measurements.** To test the coherence of the last comb states in Figs. 3e, 3f, and 3g from the main manuscript, we selected a subset of the comb lines of each comb, conducted line-by-line pulse shaping experiments, and measured the autocorrelation [1]; **a, b,** and **c** are the corresponding autocorrelation results, respectively. Green and blue curves in each figure are the experimentally measured autocorrelation traces before and after the phase correction, respectively, and red is the calculated autocorrelation that is based on the measured power spectrum and assuming flat spectral phase. In **a** and **c**, notice the clear difference between before (green) and after (blue) the phase correction; the phase corrected results (blue) are close to the calculated autocorrelations (red). These data indicate the coherence of the last comb states in Figs. 3e and 3g, as we anticipated from the low radio-frequency (RF) noise [1]. However, in **b**, a clear difference between the phase corrected (blue) and calculated (red) autocorrelations remain, even with the phase correction via line-by-line shaping. In particular, the experimental autocorrelation trace shows very poor contrast ratio compared both to the calculated curve and to the autocorrelation traces in **a** and **c**. Poor contrast ratio in intensity autocorrelation is a hallmark of broadband intensity noise [1, 3]. Thus, the autocorrelation data indicate that the coherence of the last comb state in Fig. 3f is poor, consistent with the high RF noise observed for this comb.

SUPPLEMENTARY REFERENCES

[1] Ferdous, F. *et al.* Spectral line-by-line pulse shaping of on-chip microresonator frequency combs. *Nat. Photon.* **5**, 770–776 (2011).

[2] Herr, T. *et al.* Universal formation dynamics and noise of kerr-frequency combs in microresonators. *Nat. Photon.* **6**, 480–487 (2012).

[3] Weiner, A. *Ultrafast optics*, vol. 72 (John Wiley & Sons, 2011).
